# Gallic Acid Inhibited Matrix Invasion and AP-1/ETS-1-Mediated MMP-1 Transcription in Human Nasopharyngeal Carcinoma Cells

**DOI:** 10.3390/ijms18071354

**Published:** 2017-06-24

**Authors:** Jong-Hwei S. Pang, Jia-Hau Yen, Hsiao-Ting Wu, Sheng-Teng Huang

**Affiliations:** 1 Graduate Institute of Clinical Medical Sciences, College of Medicine, Chang Gung University, Tao-Yuan City 333-02, Taiwan; jonghwei@mail.cgu.edu.tw (J.-H.S.P); b8606059@tmu.edu.tw (H.-T.W.); 2 Department of Physical Medicine and Rehabilitation, Chang Gung Memorial Hospital, Tao-Yuan City 333-02, Taiwan; 3 Research Center for Traditional Chinese Medicine, Department of Medical Research, China Medical University Hospital, Taichung 404-02, Taiwan; hugtrghu@yahoo.com.tw; 4 Department of Chinese Medicine, China Medical University Hospital, Taichung 404-02, Taiwan; 5 School of Chinese Medicine, China Medical University, Taichung 404-02, Taiwan

**Keywords:** gallic acid, invasion, MMP-1, AP-1, ETS-1

## Abstract

Gallic acid is a trihydroxybenzoic acid found in natural herbal plants. Gallic acid has been reported to inhibit the migration and invasive capability of various cancers. Little is known about the underlying mechanisms of invasion responsible for cancer metastasis via gallic acid. The present study was intended to investigate the anti-invasive effect of gallic acid on human nasopharyngeal carcinoma cells (NPC-BM1) and its related mechanism. Gallic acid inhibited the invasion of NPC-BM1 cells dose- and time-dependently without significant cytotoxic effect. Affymetrix oligonucleotide microarray analysis revealed matrix metalloproteinase-1 (MMP-1) as the most down-regulated gene in NPC-BM1 cells by gallic acid. The cytosolic and secreted MMP-1 levels were both found to be inhibited by gallic acid as demonstrated by western blot analysis and ELISA respectively. The mRNA expression and transcription of MMP-1 gene was also down-regulated as determined by RT/real-time PCR and promoter activity assay. The expression of two major transcription binding factors in the MMP-1 promoter, AP-1 and ETS-1, were demonstrated to be reduced by gallic acid in NPC-BM1 cells. The effect of gallic acid was associated with the inhibition of p38 MAPK signaling pathway. In addition, gallic acid enhanced the gene expression of tissue inhibitor of matrix metalloproteinase-1 (TIMP-1) which further suppressed the MMP-1 activity. These findings may be useful to develop a novel chemotherapeutic agent to inhibit the metastasis of nasopharyngeal cancer.

## 1. Introduction

Gallic acid is a trihydroxybenzoic acid commonly present in many natural herbal plants and also in some prescribed Chinese medicines as an active component [1,2,3,4]. This compound has several important pharmacological properties including anti-inflammatory, anti-tumor, anti-mutagenic, anti-allergic, antiulcer, antioxidant, and antiviral activities [5,6]. In our previous studies, *Phyllanthus urinaria* (*P. urinaria*), a gallic acid-containing folk herbal medicine in Taiwan, has been used to treat various cancers. The anti-cancer activity of *P. urinaria* extract is mainly due to the induced apoptosis of cancer cells as demonstrated by DNA fragmentation and increased caspase-3 activity through both intrinsic and extrinsic pathways [7,8]. In addition, *P. urinaria* also exhibits anti-angiogenic activity that is mediated by the suppressed secretion of matrix metalloproteinase 2 (MMP-2) and direct inhibition of MMP-2 activity through zinc chelation [4]. Three major compounds in *P. urinaria* were identified to be corilagin, gallic acid and ellagic acid. Ellagic acid has been demonstrated in our published studies to contribute to the anti-angiogenic effect of *P. urinaria* also by reducing MMP-2 secretion and inhibiting MMP-2 activity [3,4].

Gallic acid is known to exert anti-cancer effect. In recent years, gallic acid has been shown to induce cell death of various cancer cells originated from cervical cancer, hepatocellular carcinoma, colon cancer, non-small cell lung cancer, oral cancer and lymphoblastic leukemia, etc. [1,6]. The underlying mechanisms involve GSH depletion, ROS-dependent mitochondria activation or the blocking of EGFR signal pathway [9,10]. Gallic acid has been found in our previous study to reduce the number of viable nasopharyngeal carcinoma (NPC-BM1) cells with underlying mechanism not yet understood [8]. Gallic acid has been reported to inhibit the migration and invasive capability of prostate cancer cells, melanoma cells and gastric adenocarcinoma cells, possibly through suppressing the expression of MMP-2 and MMP-9 and the related upstream signaling pathways [11,12,13]. Although the basement membrane-degrading enzymes, MMP-2 and MMP-9, have been given considerable attention for their roles in invasion and metastasis, the interstitial collagenases, a subfamily of MMPs that cleaves the stromal collagens types I and III, have received relatively little recognition for their part in these processes. This subfamily covers collagenase 1 (MMP-1), collagenase 3 (MMP-13), and the MT-MMPs, membrane-bound MMPs to function not only for extracellular matrix remodeling during organ development and tissue regeneration but also in many pathological situations and tumor progression and metastasis. These collagenases are regulated by extracellular signals via cellular signal transduction pathways at transcriptional and post-transcriptional level to express their potentially destructive characteristics. Nasopharyngeal carcinoma is an aggressive tumor with a significantly high percentage of patients with distant metastasis which badly complicates the treatment. The induction of MMP-1 has been shown to contribute to the invasion and metastasis of NPC-BM1 [14], suggesting MMP-1 might be a new target for developing therapeutic drug. The present study, for the first time, investigated the anti-invasive effect of gallic acid on NPC-BM1 cells and focused on the regulation of MMP-1 gene expression and related signaling pathway. The expression of tissue inhibitor of metaloprotenase-1 (TIMP-1) which directly inhibits MMP-1 activity was also examined. 

## 2. Results

### 2.1. Gallic Acid Suppressed the In Vitro Matrix Invasion of NPC-BM1 Cells

**Figure 1:**
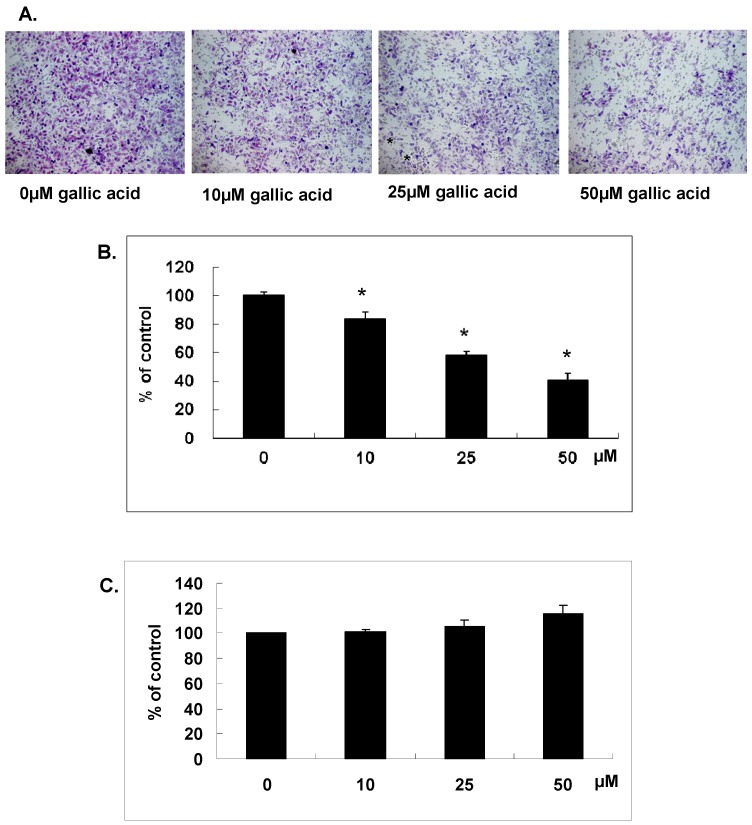
Gallic acid suppressed the in vitro matrix invasion. NPC-BM1 cells pretreated 24 h with different concentrations of gallic acid were allowed to invade for 2 h by transwell filter assay. (**A**) The cells on the lower surface of the filter were examined under contract microscope (HPF) (100×); (**B**) The inhibitory effect of gallic acid on NPC-BM1 invasion was demonstrated; (**C**) The cytotoxic effect of gallic acid was evaluated by the LDH assay using the conditioned medium collected from NPC-BM1 treated with different concentrations of gallic acid for 24 h. There was no cytotoxicity detected under the experimental conditions. Data were mean ± SD calculated from three individual experiments. An asterisk demonstrated the significant difference (* *p* < 0.05) compared with control.

As shown in our previous study, the number of viable NPC-BM1 cells was reduced by gallic acid treatment in a dose-dependent manner. To further study the anti-invasive effect of gallic acid on NPC-BM1 cells, the invasive ability of NPC-BM cells with or without gallic acid pretreatment was analyzed in the matrigel coated chamber for 24 h. Results demonstrated that the invasive ability of NPC-BM1 was inhibited by gallic acid in a dose-dependent manner (Figure 1A). Gallic acid treatment at 25 µM could inhibit half of the invasive ability in comparison with untreated control cells (Figure 1B). To rule out the possibility that this result might be simply due to the cytotoxicity of gallic acid on NPC-BM1 cells, the LDH activity in conditioned medium was measured and result confirmed no cytotoxic effect as shown in Figure 1C. The result clearly proved that gallic acid could significantly inhibit the process of migration and matrix invasion of NPC-BM1 cells. 

### 2.2. Gallic Acid Reduced MMP-1 Expression in NPC-BM1 Cells

**Figure 2:**
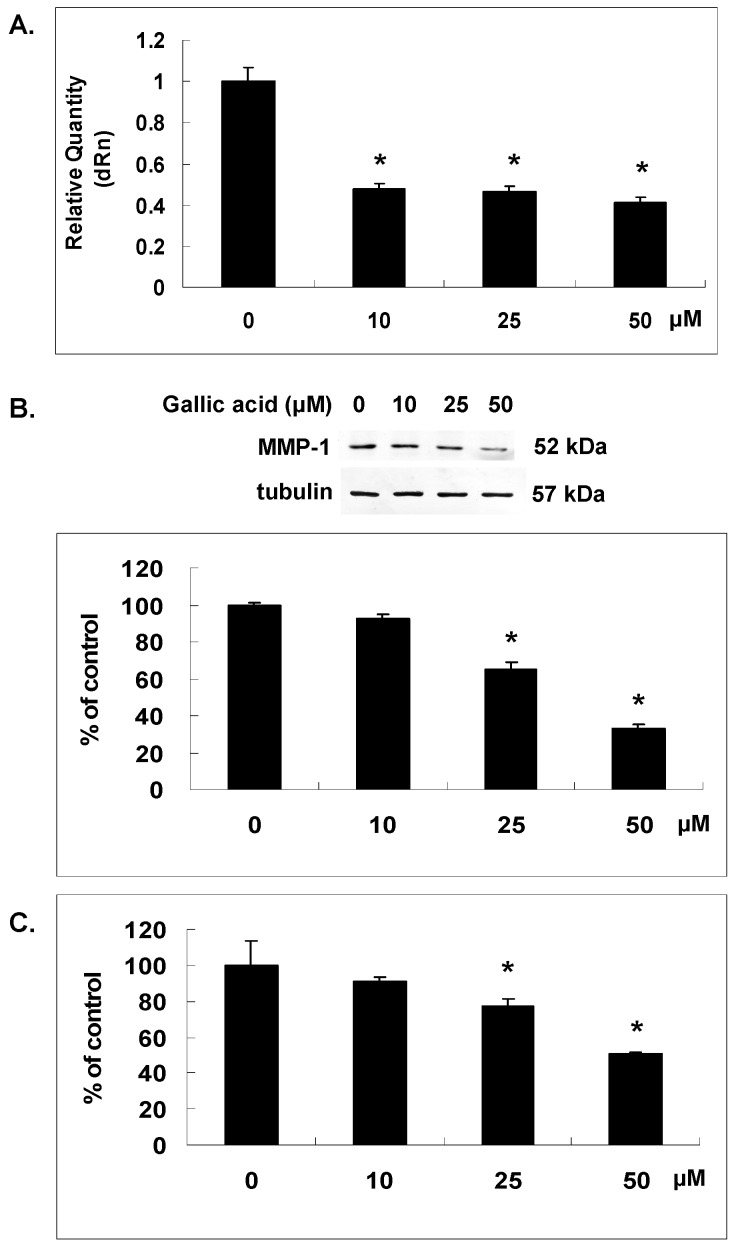
Gallic acid reduced matrix metalloproteinase-1 (MMP-1) expression**.** NPC-BM1 cells were treated with gallic acid for 24 h and the expressions of MMP-1 related to the mRNA (**A**); protein in cytosol (**B**) and conditioned medium; (**C**) were measured by qRT–PCR, Western blot and ELISA analysis respectively. The gallic acid dose-dependently decreased MMP-1 gene and protein expressions in NPC-BM1 cells. Delta Rn (dRn) indicated the magnitude of fluorescence signal generated during the PCR at each time point. Data were representative results from experiments repeated at least three times. * *p* < 0.05 versus vehicle control.

In order to further understand the potential mechanism underlying the inhibitory effect of gallic acid on the invasion of NPC-BM1 cells through matrix, microarray was performed to reveal the change of gene expression in gallic acid-treated NPC-BM1 cells. Data analysis of cDNA microarray revealed a significant change of gene expression profile in NPC-BM1 cells after the treatment with gallic acid for 24 h (Table 1 and Table 2). Among these genes, MMP-1 was the most down-regulated gene in NPC-BM1 cells affected by gallic acid. We further confirmed the inhibitory effect of gallic acid on MMP-1 mRNA expression by RT/real-time PCR (Figure 2A). The protein level of MMP-1 in cytosol and conditioned media was analyzed by western blot and ELISA as shown in Figure 2B,C, respectively. Results confirmed that gallic acid dose-dependently decreased MMP-1 gene and protein expressions in NPC-BM1 cells. 

### 2.3. Gallic Acid Inhibited MMP-1 Promoter Activity 

**Figure 3:**
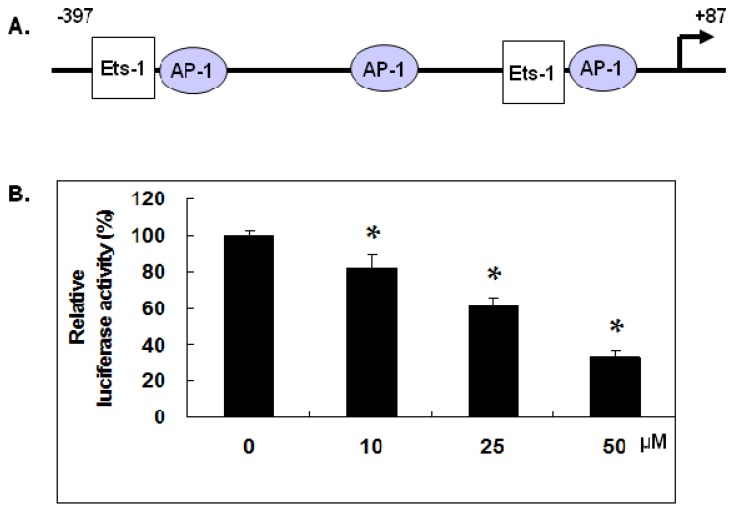
Gallic acid inhibited MMP-1 promoter activity. (**A**) The diagram of MMP-1 promoter containing the AP-1 and Ets-1 binding motifs within the –397 to +87 bp region; (**B**) The MMP-1 promoter activity in NPC-BM1 cells with or without treatment of gallic acid was detected by the dual luciferase assay. The gallic acid dose-dependently decreased the MMP-1 promoter activity in NPC-BM1 cells compared to the control. Data were representative results from experiments repeated at least three times. * *p* < 0.05 versus vehicle control.

The mRNA expression of MMP-1 gene is known to be regulated at the promoter region by transcription factors including AP-1 and Est-1. We have constructed the −397 to +87 bp region of the MMP-1 promoter containing the AP-1 and Ets-1 binding motifs in pGL3 vector as shown in Figure 3A and dual luciferase assay was performed to measure the MMP-1 promoter activity in NPC-BM1 cells with or without treatment of gallic acid. Results demonstrated that gallic acid at 10, 25 and 50 µM could dose-dependently decrease the MMP-1 promoter activity in NPC-BM1 cells by approximate 20%, 40% and 70% ,respectively, in comparison with control level as shown in Figure 3B. 

### 2.4. Gallic Acid Inhibited the AP-1 Expression and p38 MAPK Activation

**Figure 4:**
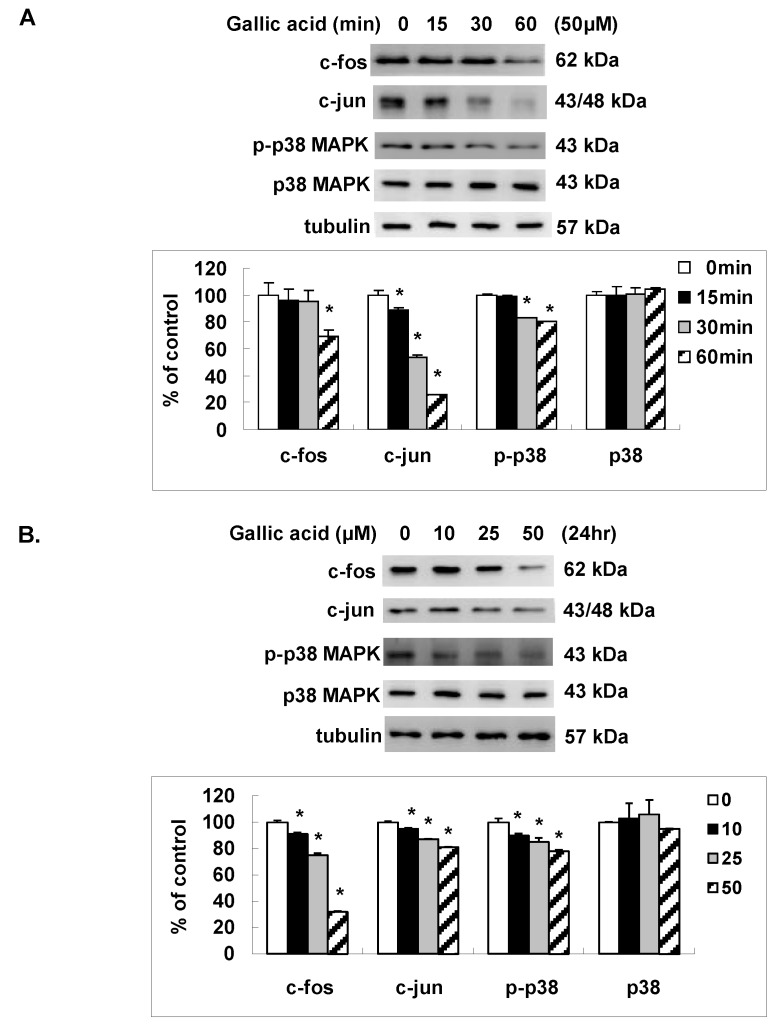
Gallic acid inhibited the AP-1 expression and p38 MAPK activation. Gallic acid inhibited the expression of c-fos, c-jun and the activation of p- p38 MAPK in NPC-BM1 cells with time- (**A**) and dose- (**B**) dependent manner by Western blot. Data were representative results from experiments repeated at least three times. * *p* < 0.05 versus vehicle control.

To elucidate the effect of gallic acid on the AP-1 transcription factor that might result in the decrease of MMP-1 promoter activity, we analyzed the expressions of c-fos and c-jun, two distinct components of AP-1, by western blot. As shown in Figure 4A,B, gallic acid time- and dose- dependently decreased the protein expression of c-jun and c-fos in NPC-BM1 cells. To determine if p38 MAPK phosphorylation, the upstream signal pathway of AP-1 transcription factor was affected by gallic acid, we also analyzed the protein expressions of p38 and phosphorylated p38 in NPC-BM1 cells after gallic acid. As shown in Figure 4A,B, the activation of p38MAPK signal pathway was inhibited in parallel with the down-regulation of c-fos and c-jun in NPC-BM1 cells by gallic acid. 

### 2.5. Gallic Acid Inhibited the Est-1 Expression in NPC-BM1 Cells

**Figure 5:**
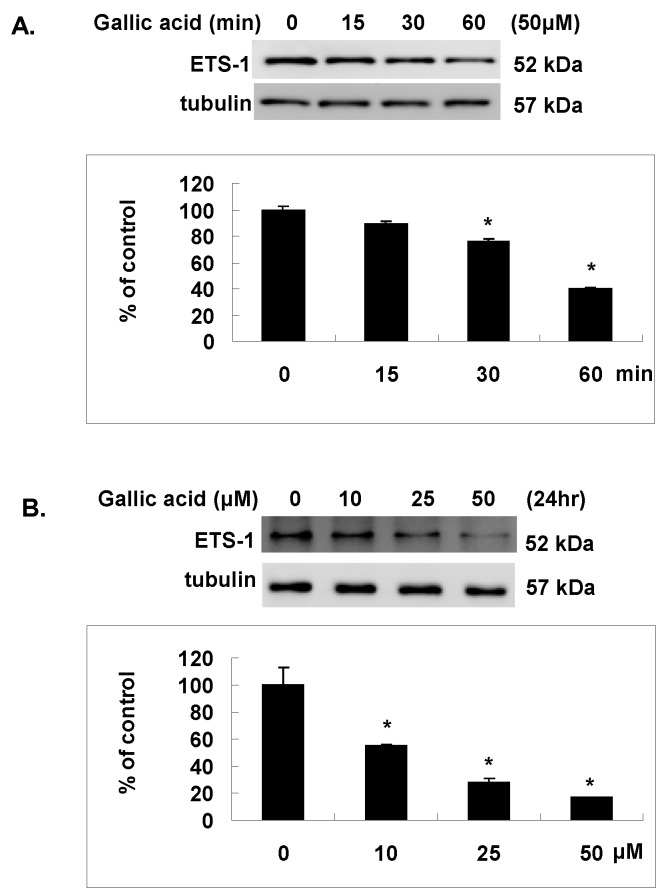
Gallic acid inhibited the Est-1 expression. Gallic acid inhibited the ETS-1 activation in NPC-BM1 cells with time- (**A**) and dose- (**B**) dependent manner by Western blot. Data were representative results from experiments repeated at least three times. * *p* < 0.05 versus vehicle control.

Except AP-1, Est-1 is another transcription factor that acts synergistically with AP-1 to regulate the transcription of MMP-1 gene with their binding sites located within the proximal region of MMP-1 promoter as shown in Figure 3A. To elucidate the effect of gallic acid on the Est-1 transcription factor that might also cause the inhibition of MMP-1 promoter activity, we analyzed the expressions of Est-1 by western blot. As shown in Figure 5A,B, gallic acid could also time- and dose- dependently decrease the protein expression of Est-1 in NPC-BM1 cells.

### 2.6. Gallic Acid Increased the TIMP-1 Expression in NPC-BM1 Cells

**Figure 6:**
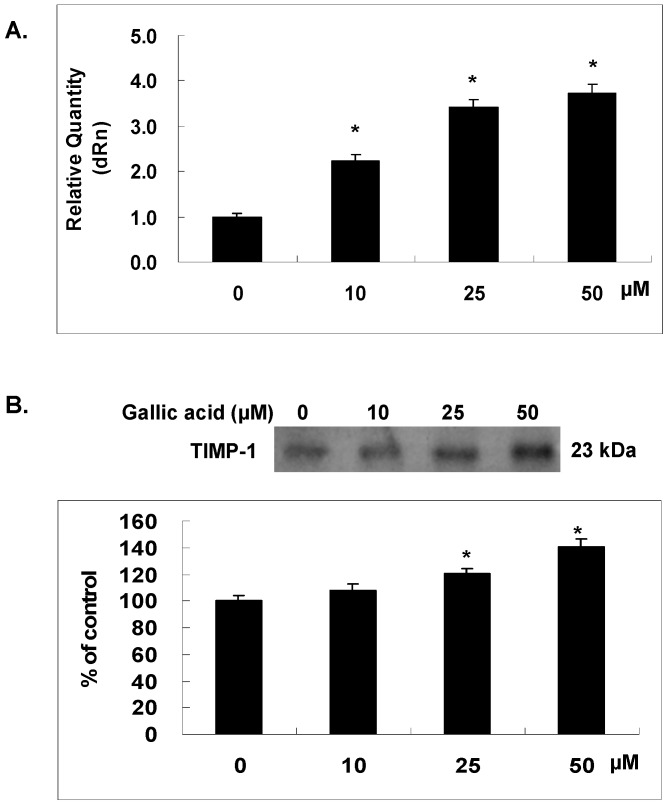
Gallic acid increased the TIMP-1 expression. NPC-BM1 cells were treated with gallic acid for 24 h and the expression levels of TIMP-1 mRNA (**A**) and protein in condition medium (**B**) were analyzed by qRT–PCR and Western blot respectively. The gallic acid dose-dependently increased TIMP-1 gene and protein expressions in NPC-BM1 cells. Delta Rn (dRn): The magnitude of the fluorescence signal generated during the PCR at each time point. Data were representative results from experiments repeated at least three times. * *p* < 0.05 versus vehicle control.

TIMP-1, a tissue inhibitor of metalloproteinase counteracts with MMP-1 to modulate the tissue destruction and balance the extracellular matrix. To test the hypothesis that MMP-1 and TIMP-1were both regulated by gallic acid and contributed to the inhibited matrix invasion of NPC-BM1 cells, we examined the mRNA and protein expression of TIMP-1 in NPC-BM1 cells after gallic acid treatment for 24 h by RT/real-time PCR and western blot, respectively. As shown in Figure 6, gallic acid increased the mRNA expression of TIMP-1 in NPC-BM1 cells (Figure 6A). The protein level of TIMP-1 in conditioned media of NPC-BM1 cells was also enhanced by gallic acid dose-dependently as shown in Figure 6B. The increase of TIMP-1 further rendered the MMP-1 activity more inactive.

## 3. Discussion

Nasopharyngeal carcinoma, a common head and neck malignant tumor arises from the lining of the nasopharynx with the higher prevalence in Southern China and Africa. Combined therapy has significantly improved the 5-year survival of NPC-BM1 patients. However, nearly 30% of NPC-BM1 patients eventually developed treatment failure due to distant metastasis [15]. In the present study, we investigated the effect of gallic acid, a major active component of *Phyllanthus urinaria*, on the matrix invasion of NPC-BM1 cells and related molecular mechanism. Our results demonstrated that gallic acid dose-dependently decreased the matrix invasion of NPC-BM1 cells, suggesting the potential of gallic acid to reduce the chance of metastasis. Gallic acid has been shown in our previous study to decrease the number of viable nasopharyngeal carcinoma cells in a dose-dependent manner [8]. Therefore, LDH assay was carried out to exclude the cytotoxic effect of gallic acid and the invasion result was further ensured by using same number of viable cells in each of the transwell filter assay. In addition, the protein levels measured in the conditioned medium by ELISA or western blot analysis were all adjusted by the number of viable cells in each condition to verify the results.

Matrix metalloproteinases (MMPs) induced by a variety of external stimuli such as cytokines and growth factors play essential roles in ECM degradation during physiological normal tissue remodeling and in pathological tumor progression related to hyperplasia, angiogenesis, migration, and invasion [16]. The MMPs are a family of zinc dependent endopeptidases categorized as gelatinases, collagenases, stromelysins and matrilysins [3,4]. To date there are at least 28 MMPs identified with fourteen implicated in cancer development and progression [17]. MMPs activity is specifically inhibited by tissue inhibitors of metalloproteinases (TIMPs). The balanced interaction of MMPs with TIMPs regulates ECM homeostasis. The imbalance between MMPs and TIMPs is an important step in the development of malignancies. Among various MMPs that have been associated with the progression and metastasis of nasopyaryngeal carcinoma, MMP-2 and MMP-9 have been reported up-regulated in blood and malignant tissue of NPC-BM1 patient in many studies [18]. Recently, MMP-1 was reported to be remarkably expressed in NPC-BM1 biopsies and the up-regulation of MMP-1 has been demonstrated to be correlated with lymph node metastasis of NPC-BM1 [14,19]. EBV is known to strongly associate with NPC-BM1 growth. Study has shown clearly that the amounts of transcripts, proteins, and enzyme activities of MMP-1 were specifically up-regulated in cells expressing EBV proteins. Furthermore, anti-MMP-1 antibody and peptide inhibitors of MMP-1 could block the invasiveness and survival properties of these cells, suggesting an important contribution of MMP-1 to NPC-BM1 progression [20]. Because NPC-BM1 expresses more MMP-1 than other head and neck cancers, MMP-1 may contribute to the unique high metastatic rate of NPC-BM1. The up-regulation of MMP-1 in combination with PAR-1 overexpression is also an unfavorable prognostic marker for NPC-BM1 which might offer the possibility of future therapeutic targets [14]. Microarray analysis in the present study revealed MMP-1 as the most down-regulated gene in NPC-BM1 cells treated by gallic acid, which was further confirmed by RT/real-time PCR, western blot analysis and ELISA. Gallic acid has been shown to regulate MMP-1 gene expression, however, only in skin cells such as epidermal keratinocyte and dermal fibroblast [21]. Our study, for the first time, demonstrated the inhibitory effect of gallic acid on the gene expression of MMP-1 in NPC-BM1 cells. We further confirmed that gallic acid promoted the expression of TIMP-1 at both the mRNA and protein levels, indicating an enhanced effect of gallic acid to suppress MMP-1 activity.

The gene expression of MMP-1 is complexly regulated at different levels in which the activation of promoter and transcription is known to play a major role. The promoter polymorphisms of MMP-1 have also been implicated in increased susceptibility of nasopharyngeal carcinoma [22]. Our study demonstrated the inhibitory effect of gallic acid on the transcriptional activity of MMP-1 promoter region spanning from −397 to +87 bp in NPC-BM1 cells. Analysis of the −397 to +87 bp region for putative transcription binding sites revealed the presence of two Ets-1 binding sites and three AP1 binding sites. Trans-activation by Ets-1 and AP1 is synergistic, and mutation of the individual binding sites reveals that the transcriptional activities of these factors are inter-dependent [23]. Ets-1 has been shown to highly express within the malignant keratinocytes and correlate with the invasive and metastatic potential of the tumor and linked to the pathogenesis of squamous cell carcinoma. Ets-1 is also expressed only in the basal layer of stratified squamous epithelium of normal tissue and sufficient to induce invasion especially for oral cancer [24]. According to the World Health Organization classification, nasopharyngeal carcinoma includes three types. Type 1 (I) is squamous cell carcinoma. Type 2a (II) is keratinizing undifferentiated carcinoma. Type 2b (III) is nonkeratinizing undifferentiated carcinoma [25] (p. 1344). Using Western blot analysis and qRT-PCR, we further identified that gallic acid inhibited the expression of Ets1 at both the mRNA and protein levels, indicating the reduction of critical transcription factor for the activation of MMP-1 transcription. LMP1 induces transcriptional activation and expression of Ets-1 which may contribute to the development and metastasis of NPC-BM1. LMP1 up regulates 3′E(κ) activity and κ gene expression by activating the Ets-1 transcription factor through the ERKs signaling pathway. The blocking of SSRP1/Ets-1/Pim-3 signaling in NPC-BM1 cells also facilitates chemosensitivity of the cells to docetaxel [26]. In addition, it has been reported that MMP-9 gene promoter contains a binding site for Ets-1 in addition to AP-1 and NF-κB [27]. Therefore, the inhibition of Ets-1 by gallic acid might also modulate the MMP-9 expression which further contributes to the suppression of NPC-BM1 progression and invasion. 

MMP-1 has been well studied in several systems in which the interaction of promotor with the AP-1 proteins, c-Fos and c-Jun has often been implicated in tumorigenesis [28,29,30,31]. Transcription from both the c-Fos and c-Jun genes is rapidly and transiently induced in cells that are treated with serum or peptide growth factors via oncogene mediated signal transduction networks. The permutations of hetero- and homodimer formation among c-Fos and c-Jun family members with the known interactions of AP-1 with other transcription factor families can exert a finely tuned regulation of large sets of genes in response to a wide variety of extracellular and intracellular stimulation [32]. Our results demonstrated that the expression of both c-Jun and c-Fos in NPC-BM1 cells was dose-dependently inhibited by gallic acid. Inhibition of c-Jun and c-Fos eventually resulted in the down-regulation of MMP-1 gene transcription. The EBV-encoded EBNA1 protein is known to play an essential role in EBV genome maintenance, replication and transcription. In addition, EBNA1 has also been found to elevate the levels of VEGF and the phosphorylated isoforms of c-Jun and ATF2 in NPC-BM1 biopsies and involve in the angiogenic process contribute to the development and aggressively metastatic nature of NPC-BM1. AP-1 appears to be a novel target for treating or preventing LMP1-positive NPC-BM1 effectively. Therefore, the inhibitory effect of gallic acid on decreasing the expression of c-Fos and c-Jun is quite important. 

Angiogenesis is known to be absolutely required in cancer development for oxygen and nutrient supply [33]. The high expression of matrix metalloproteinases (MMPs) is correlated with the growth and metastasis of preexisting tumors. MMPs play an important role on promoting tumor growth and increasing endothelial cell permeability, migration and invasion by regulating angiogenic factors including bFGF, VEGF and PDGF [34]. Gallic acid has been shown to inhibit VEGF secretion and suppress in vitro angiogenesis in a concentration-dependent manner. The luciferase assay results suggest that the PTEN/AKT/HIF-1α pathway accounts for the inhibitory effect of GA on VEGF expression and in vitro angiogenesis [35]. Since angiogenesis is an essential component of the metastatic pathway, the anti-angiogenic effect of gallic acid may exert better therapeutic effect on tumor development and metastasis.

## 4. Materials and Methods

### 4.1. Materials

Gallic acid, ECM gel was purchased from Sigma (St. Louis, MO, USA). Monoclonal antibodies against MMP-1, TIMP-1, and tubulin were obtained from Neomarker (Fremont, CA, USA). Monoclonal antibodies against c-fos, c-jun, p-p38 and polyclonal antibodies against p-38, ETS-1 were obtained from Cell Signaling Technology (Beverly, MA, USA).

### 4.2. Cell Culture

The human nasopharyngeal carcinoma cells (NPC-BM1), was obtained from Shuen-Kuei Liao at Taipei Medical University (Taipei, Taiwan) and grown in an adhesion culture in DMEM medium supplemented with 10% (*v*/*v*) fetal bovine serum and antibiotics. Cells were subcultured in a 1:3 ratio and grown at 37 °C in a humidified atmosphere with 5% CO_2_/95% air. Cells were grown to 70–80% confluence before treatment.

### 4.3. Cell Invasion Assay

Cell invasion assays were performed using transwell filters with 8-µm pore size (Corning Inc., Corning, NY, USA). A 50-μg aliquot of matrigel solution was placed on the filter surface and air dried overnight to produce an artificial basement membrane. Cells were washed once with PBS and 1.5 × 10^5^ cells in 200 µL DMEM were added to the upper chamber and 600 µL DMEM containing 10% FBS, in the lower chamber. NPC-BM1 cells were treated with 0, 10, 25 and 50 µΜ gallic acid for 24 h. The invasion was carried out for 24 h and NPC-BM1 cells on the filter were first stained with Liu’s stain and cells that remained on the upper surface of the filter were removed using a cotton swab. The cells that migrated onto the lower surface of the filter were examined by microscope after mounting on a slide. A total of six random high-power microscopic fields (HPF) (100×) per filter were photographed and the numbers of cells were directly counted.

### 4.4. Cytotoxicity Assay

Lactate dehydrogenase (LDH) activity was measured using the CytoTox 96^®^ Non-Radioactive Cytotoxicity Assay (Promega, Mannheim, Germany). Disruption of plasma membrane integrity leads to a release of LDH into the supernatant and results in the conversion of a tetrazolium salt into a red formazan product. NPC-BM1 cells were treated with various concentrations of gallic acid for 24 h and 50 µL of the supernatant was collected for assay. The LDH activity was measured and read at 490 nm using a spectrophotometer.

### 4.5. Microarray Analysis 

The GMRCL Human 7K set, Version 2 chips were used for the analysis of gene expression profiles. RNA was isolated from NPC-BM1 cells with or without 50 μM gallic acid treatment for 24 h. Bioanalyzer 2100 (Agilent, Santa Clara, CA, USA) was used to evaluate RNA quality and quantity. Fluorescent labeling of target mRNA was prepared by indirect 3DNA Submicro Expression Array Detection kit (Genisphere, Hatfield, PA, USA). RNA was reverse transcribed to target cDNA using oligo-d(T) primer tagged with either Cys-3 or Cys-5-specific 3 DNA-capture sequences and hybridized to microarray for 16 h. Next step, the in situ labeling with Cys-3DNA or Cys-5-3DNA based on the sequence-complementation to the capture sequences was carried out for 2 h. After washing, slides were scanned with a confocal scanner ChipReader (Virtek Vision International Inc., Waterloo, ON, Canada). We acquired the spot and background intensities with GenePix Pro 4.1 software (Axon Instruments/Molecular Devices Corp., Sunnyvale, CA, USA) and carried out within-slide normalization with programs written by MATLAB 6.0 software (The MathWorks, Inc., Natick, MA, USA). 

### 4.6. RNA Isolation

Total cellular RNA was isolated by lysis in a guanidinium isothiocyanate buffer, followed by single-step phenol-chloroform-isoamyl alcohol extraction. Briefly, 5 × 10^6^ cells were lysed in 0.5 mL solution D containing 4 M guanidinium isothiocyanate, 25 mM sodium citrate (pH 7.0), 0.5% sodium sarcosine, and 0.1 M β-mercaptoethanol with vigorous vortex. Sequentially, 50 µL of 2 M sodium acetate (pH 4.0), 0.5 mL of phenol, and 100 µL of chloroform: isoamyl alcohol (49:1, *v*:*v*) were added to the homogenate. After vortex for 30 s, the solution was centrifuged at 10,000× *g* for 15 min at 4 °C. The RNA was precipitated by the addition of 0.5 mL isopropanol and kept at −20 °C for 1 h. RNA was pelleted by centrifuging the solution at 10,000× *g* for 15 min at 4 °C. After the RNA pellet was rinsed in ice-cold, 75% ethanol, the dry RNA was dissolved in DEPC-treated ddH_2_O.

### 4.7. Real-Time RT-PCR

The cDNA was synthesized from total RNA using M-MLV reverse transcriptase (USB Corporation, Cleveland, OH, USA). Quantitative real time PCR was performed with universal cycling conditions (10 min at 95 °C, 40 cycles of 30 s at 95 °C, 1 min at 55 °C, and 30 s at 72 °C). Cycle threshold values were determined by automated threshold analysis with Mx-Pro Mx3005P v4.00 software (Agilent Tech, Santa Clara, CA, USA). The PCR primers used were as the followings: MMP-1 forward primer, 5′-TTCCACAGGTCCCACAAC-3′, and reverse primer, 5′-GCATTCCTCACAGCCAAC-3′; TIMP-1 forward primer, 5′-CAGTAGAATGGGAGAGTC-3′, and reverse primer, 5′-GGTGATGAAGCAGCCCAG-3′; and GAPDH forward primer, 5′-GACCTGACCTGCCGTCTA-3′, and reverse primer, 5′-AGGAGT GGGTGTCGCTGT-3′. The mRNA expression levels were normalized to the amount of GAPDH mRNA. To calculate gallic acid-induced changes in mRNA levels, relative mRNA expression was related to that of the control which was set to 1.0.

### 4.8. Western Blot Analysis

Cell extracts were prepared in lysis buffer containing Tris-HCl, pH 7.5, 150 mM NaCl, 1 mM EDTA, 2 mM DTT, 2 mM PMSF, and 1% TritonX-100 by sonication method. The protein concentration of the cell extracts was determined using the Bradford assay (Bio-Rad Laboratories, Hercules, CA, USA). Samples with same amount of proteins were then separated by 10% SDS polyacrylamide gel electrophoresis and transferred onto a PVDF membrane. The membrane was incubated at room temperature in blocking solution containing 1% bovine serum albumin (BSA) and 1% goat serum in PBS for 1 h, followed by 2 h of incubation in blocking solution containing appropriate dilution of primary antibodies including anti-c-jun (1:1000), anti-c-fos (1:1000), anti-p38 (1:1000), anti-p-p38 (1:1000), anti-ETS-1 (1:1000), anti-MMP-1 (1:100), anti-TIMP-1 (1:100), or anti-tubulin (1:800) antibodies, After washing 3 times in PBS, the membrane was then incubated in PBS containing goat anti-mouse IgG conjugated with horseradish peroxidase (Sigma, St. Louis, MO, USA) or goat anti-rabbit conjugated with horseradish peroxidase (Cell Signaling, Boston, MA, USA) for 1 h Membranes were washed 3 times in PBS and positive signals were developed with enhanced chemiluminescence (Amershan Pharmacia Biotech, Little Chalfont Buckinghamshire, England).

### 4.9. ELISA Assay

A human MMP-1-specific ELISA kit (R&D Systems, Minneapolis, MN, USA) was used to determine the levels of MMP-1 in conditioned media collected from NPC-BM1 cells. The experimental steps were carried out as described in the protocol provided by manufacturer. This study was performed three times and each time was analyzed in duplicate.

### 4.10. Plasmid Construction

A 484bp (−397 to +87) segment at the 5′ flanking region of the human MMP-1 gene was amplified by PCR using specific primers from the human MMP-1 gene: 5′-GCCACCGTAAAGTGAGTG-3′ (sense) and 5′-TTGCTGCTCCAATATCCC-3′ (anti-sense). The PCR product was TOPO cloned into the pCR2.1 plasmid (Invitrogen, Carlsbad, CA, USA) using the manufacturer’s instructions. The SacI and XhoI fragments digested from the cloned PCR product of human MMP-1 promoter were subcloned into the luciferase reporter vector pGL3-Basic (Promega, Madison, WI, USA) and used for the analysis of MMP-1 promoter activity.

### 4.11. Transient Transfection and Dual Luciferase Assay

The plasmid pGL3-Basic or pGL3-MMP-1 promoter was co-transfected with pRL-TK into nasopharyngeal carcinoma cells using Arrest-In Transfection Reagent (Thermo, Waltham, MA, USA) Two different luciferase activities were then measured 24 h after the transfection using Dual-Glo^®^ Luciferase Assay System (Promega). After subtracting the background value, data were normalized to the activity of Renilla Luciferase before statistical analyses.

### 4.12. Statistical Analysis

All statistical analyses were performed using SigmaStat statistical software (version 2.0, Jandel Scientific, San Rafael, CA, USA). Results were represented as means ± standard deviation. One-way ANOVA was carried out when multiple comparisons were evaluated. Values were considered to be significant at *p* less than 0.05. All experiments were repeated at least three times independently.

## 5. Conclusions

Dietary polyphenols have been demonstrated to exhibit both anti-cancer [36] and anti-angiogenic [37] effects. Gallic acid, a naturally occurring low molecular weight triphenolic compound among various polyphenols, has been investigated in the present study. We firstly demonstrate that gallic acid can transcriptionally suppress matrix metalloproteinase-1 (MMP-1) expression through the down-regulation of Ets1 and c-Jun, c-fos of the AP-1 family. It is also for the first time to show that gallic acid can significantly promote the expression of TIMP-1 which further inhibits the MMP-1 activity by direct binding to active site. The fact that gallic acid inhibits the MMP-1 activity may provide a valuable pharmacological choice for treatment of nasopharyngeal cancer. However, continuous efforts are needed to address and provide critical information for the identification and validation of the in vivo study for further pharmaceutical development. 

## Figures and Tables

**Table 1:** List of genes downregulated by gallic acid in nasopharyngeal carcinoma.

Gene Description	UniGene Symbol	Fold Decrease
matrix metallopeptidase 1 (interstitial collagenase)	MMP1	2.9
interferon, α-inducible protein 27	IFI27	2.8
tripartite motif-containing 22	TRIM22	2.4
2′-5′-oligoadenylate synthetase 2, 69/71kDa	OAS2	2.4
2′,5′-oligoadenylate synthetase 1, 40/46kDa	OAS1	2.3
interferon-induced protein with tetratricopeptide repeats 1	IFIT1	2.2
keratin 13	KRT13	2.2
small proline-rich protein 1B (cornifin)	SPRR1B	2.2
lectin, galactoside-binding, soluble, 7 (galectin 7)	LGALS7	2.1
chemokine (C-C motif) ligand 5	CCL5	2.1
interferon-induced protein 44-like	IFI44L	2.0

**Table 2:** List of genes upregulated by gallic acid in nasopharyngeal carcinoma.

Gene Description	UniGene Symbol	Fold Increase
hemoglobin, α 2	HBA2	0.26
hemoglobin, α 1	HBA1	0.27
aldo-keto reductase family 1	AKR1C2	0.28
chromosome 6 open reading frame 48	C6orf48	0.32
hemoglobin, β	HBB	0.35
cytochrome P450, family 1, subfamily A, polypeptide 1	CYP1A1	0.37
lipocalin 2 (oncogene 24p3)	LCN2	0.40
N-myc downstream regulated gene 1	NDRG1	0.44
thioredoxin interacting protein	TXNIP	0.44
laminin, β 3	LAMB3	0.44
thioredoxin reductase 1	TXNRD1	0.44
hypothetical protein DJ328E19.C1.1	DJ328E19.C1.1	0.45
sialidase 1 (lysosomal sialidase)	NEU1	0.48
uridine phosphorylase 1	UPP1	0.53
calbindin 1, 28kDa	CALB1	0.54
aldolase C, fructose-bisphosphate	ALDOC	0.55
hypothetical protein MAC30	MAC30	0.57
hypothetical protein MGC14376	MGC14376	0.58
lipin 1	LPIN1	0.58

## Abbreviations

NPC	nasopharyngeal carcinoma
MMP	matrix metalloproteinase
TIMP	tissue inhibitor of matrix metalloproteinase
*P. urinaria*	*Phyllanthus urinaria*
GSH	glutathione
ROS	reactive oxygen species
EGFR	epidermal growth factor receptor
Ets-1	proto-oncogene 1
HPF	high-power microscopic field
LDH	lactate dehydrogenase
BSA	bovine serum albumin
AP-1	activator protein 1
MAPK	mitogen-activated protein kinase
ECM	extracellular matrix
EBV	Epstein-Barr virus
LMP1	latent membrane protein 1
NF-κB	nuclear factor kappa-light-chain-enhancer of activated B cells
VEGF	vascular endothelial growth factor
